# A cluster of RNA Polymerase II molecules is stably associated with an active gene

**DOI:** 10.1101/2025.02.10.637507

**Published:** 2025-02-11

**Authors:** Apratim Mukherjee, Manya Kapoor, Kareena Shankta, Samantha Fallacaro, Raymond D. Carter, Puttachai Ratchasanmuang, Yara I. Haloush, Mustafa Mir

**Affiliations:** 1 Department of Cell and Developmental Biology, Perelman School of Medicine, University of Pennsylvania; Philadelphia, PA 19104, USA.; 2 Center for Computational and Genomic Medicine, Children’s Hospital of Philadelphia; Philadelphia, PA 19104, USA.; 3 Department of Bioengineering, University of Pennsylvania; Philadelphia, PA 19104, USA.; 4 Roy and Diana Vagelos Program in Life Sciences and Management, University of Pennsylvania; Philadelphia, PA 19104, USA.; 5 Developmental, Stem Cell, and Regenerative Biology Graduate Group, Perelman School of Medicine; Philadelphia, PA 19104, USA.; 6 Biochemistry, Biophysics, and Chemical Biology Graduate Group, Perelman School of Medicine, University of Pennsylvania; Philadelphia, PA 19104, USA.; 7 Howard Hughes Medical Institute, Children’s Hospital of Philadelphia; Philadelphia, PA 19104, USA.; 8 Epigenetics Institute, University of Pennsylvania Perelman School of Medicine; Philadelphia, PA 19104, USA.

## Abstract

In eukaryotic nuclei, transcription is associated with discrete foci of RNA Polymerase II (RNAPII) molecules. How these clusters interact with genes and their impact on transcriptional activity remain heavily debated. Here we take advantage of the naturally occurring increase in transcriptional activity during Zygotic Genome Activation (ZGA) in *Drosophila melanogaster* embryos to characterize the functional roles of RNAPII clusters in a developmental context. Using single-molecule tracking and lattice light-sheet microscopy, we find that RNAPII cluster formation depends on transcription initiation and that cluster lifetimes are reduced upon transcription elongation. We show that single clusters are stably associated with active gene loci during transcription and that cluster intensities are strongly correlated with transcriptional output. Our data suggest that prior to ZGA, RNAPII clusters prime genes for activation, whereas after ZGA, clusters are composed mostly of elongating molecules at individual genes.

## Introduction

It has long been hypothesized that the organization of the nucleus into enzymatically active sub-compartments that concentrate reactants may boost the efficiency of biochemical reactions^1–4^. In the context of gene expression, this idea gained support from observations in the early 1990’s that suggested RNA synthesis and processing occurs within discrete nucleoplasmic foci^5,6^. These foci, termed transcription factories, were thought to be stable assemblies containing 10s-100s of RNAPII molecules associated with multiple transcriptionally active genes^7–9^. Super-resolution imaging in fixed cells challenged this model and suggested that transcriptional foci on average represent single RNAPII molecules^10^. Also in contrast to the transcription factory model, RNA fluorescence *in situ* hybridization imaging in fixed *Drosophila* embryos suggested that each active gene is associated with its own RNAPII cluster^11^. On the other hand, live-cell single-molecule imaging has shown that RNAPII clusters composed of multiple molecules are prevalent but are highly dynamic, lasting on the order of just seconds. These live-cell approaches suggested that cluster formation is tightly linked to transcription initiation and that cluster lifetimes determine the number of polymerases loaded onto a gene^12,13^. In contrast, recent single-molecule tracking of RNAPII suggests that clusters form post-initiation and are composed of ~6–7 elongating polymerases at a gene^14^. Given these contrasting observations made using high resolution imaging methods, it is still debated whether clusters form at initiation and dissolve prior to elongation, or only form post-initiation and represent a collection of elongating molecules at a single gene.

More recently, larger clusters of RNAPII and co-activators described as condensates, spanning half a micrometer and lasting tens of minutes have also been observed^15,16^. These condensates were initially proposed to form at super-enhancers through liquid-liquid phase-separation and transiently interact with target genes to up-regulate transcription^15^. However, subsequent studies demonstrated that their formation may not depend on super-enhancers, and that transcriptional upregulation requires condensate to gene proximities of less than a micrometer rather than direct contact^17^. Recent imaging in *Drosophila* embryos also suggests that RNAPII clusters transiently associate with active transcription sites and dissipate as nascent transcription increases^18^ consistent with models in which local RNA concentrations promote condensate formation at low levels and lead their dissipation at higher concentrations^19^. The transient associations of these larger RNAPII condensates with genes contrasts with the findings on more dynamic clusters described above. As these larger clusters have been reported to represent ~10% of the RNAPII clusters in the nuclei in which they were identified^15^, their interactions with active genes may not be representative of the majority of clusters. The nature of RNAPII cluster interactions with actively transcribing genes may thus be multifaceted and remains unclear.

Here we examine how RNAPII clustering changes in response to the sharp increase in transcriptional activity that occurs during Zygotic Genome Activation (ZGA) in *Drosophila melanogaster* embryos. Using live-embryo single-molecule tracking, lattice light-sheet microscopy, imaging of nascent transcriptional activity, and small molecule perturbations to transcription, we address mechanisms of cluster formation, changes in biophysical properties during transcriptional upregulation, and how clusters interact with active genes. We find that RNAPII cluster formation depends on transcription initiation and that their stability is reduced by transcription elongation. RNAPII clusters transition from priming individual genes for activation prior to ZGA to collections of elongating molecules after ZGA. Furthermore, we find that individual RNAPII clusters persistently associate with a single active transcription site throughout the duration of a transcription burst.

### Chromatin bound fraction of RNAPII molecules increases during Zygotic Genome Activation

**Fig. 1.**
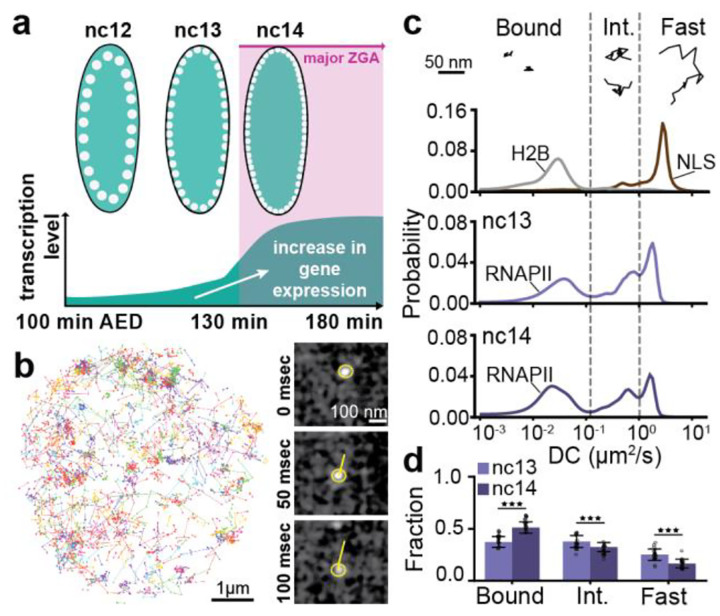
Single molecule kinetics of RNAPII during Zygotic Genome Activation (ZGA). **(a)** Timeline of zygotic genome activation (ZGA) in *Drosophila* embryos from nuclear cycles (nc) 12–14. X-axis shows minutes after egg deposition (AED). **(b)** (Left) Representative nucleus showing individual RPB1-mEos3.2 single molecule tracks over 80 sec acquired at 10 msec exposure time. (Right) Timelapse snapshots showing a single molecule (yellow circle) being tracked (yellow line) from the same nucleus over 8 frames (80 msec). **(c)** Representative individual trajectories within the different kinetic bins (bound, intermediate and fast) for RPB1. Diffusion coefficient distributions for H2B and NLS (top), RPB1 in nc13 (middle panel) and in nc14 (bottom panel). Dashed vertical lines show the division of the diffusion coefficient distributions into kinetic bins for RPB1. A total of 210,638 trajectories from 9 embryos (270 nuclei) were obtained in nc13, and 205,532 trajectories from 10 embryos (264 nuclei) in nc14, for RPB1. A total of 37,856 trajectories from 3 embryos (84 nuclei) and 8,009 trajectories from 3 embryos (76 nuclei) were obtained for H2B and NLS respectively in nc14. **(d)** Bound, intermediate, and fast fractions for RBP1 in nc13 and nc14. Each data point represents a bound fraction value obtained from 23 single fields of views (~8–15 nuclei) and error bars show standard deviations. Mann-Whitney U-test was performed to determine significance and following p-values were used: *p<0.05, **p<0.01, and ***p<0.001

The major wave of ZGA in *Drosophila* embryos occurs in the 14th nuclear cleavage cycle (nc14) during which transcription increases sharply from ~1,000 active genes before nc14, to upwards of 3,500 genes in nc14, with estimates varying by the method of quantification^20–24^ (Fig. 1a). To quantify changes in RNAPII molecular kinetics in response to this increased demand for its enzymatic activity, we endogenously tagged RPB1, the largest and catalytic subunit of RNAPII, with the photoconvertible fluorescent protein mEos3.2 (Fig. S1.1) and performed fast single molecule tracking (10 msec/frame) (Fig. 1b and Fig. S1.2a, Movie 1)^25–27^. We used state-array modeling to infer diffusion coefficients^28^ for each trajectory, and obtained distributions of diffusion coefficients for the histone H2B and nuclear localization sequence (NLS) fused to mEos3.2 in addition to RNAPII. Based on the diffusion coefficient distributions of H2B and NLS, we categorized RNAPII trajectories as (i) chromatin bound; (ii) intermediate, likely representing confined molecules or (iii) fast, freely diffusing molecules (Fig. 1c and Fig. S1.2b). We find that the fraction of chromatin-bound RNAPII molecules increases from 37±5% in nc13, to 51±8% in nc14, representing a 1.4-fold increase (Fig. 1d and Fig. S1.2c). This increase in the bound population is accompanied by a concomitant 1.6-fold decrease in the fast population and 1.2-fold decrease in the intermediate population. Overall, the change in the total nuclear RNAPII bound fraction from nc13 to nc14 is lower than expected given the known increase of at least a two-fold increase in transcriptional activity occurring over this time period^20,22,23^. To address this discrepancy, we reasoned that different steps of the transcription cycle, namely engagement, initiation, pausing, and elongation, would all appear as chromatin-bound in our data, due to our single-molecule localization error of ~30 nm. To determine changes in the fraction of RNAPII molecules engaged in each part of the transcription cycle, we turned to small-molecule inhibitors to deconvolve our chromatin-bound population^29^.

### Transcription initiation and elongation differentially contribute to the RNAPII bound fraction during ZGA

**Fig. 2.**
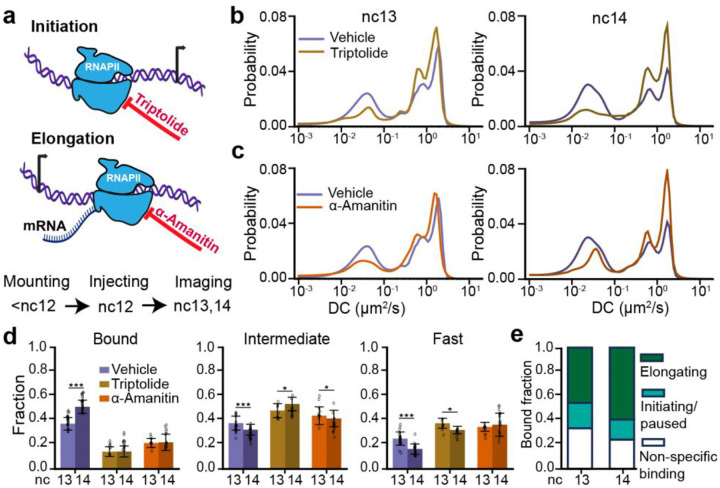
Changes in RNAPII kinetics upon inhibition of transcriptional elongation or initiation. **(a)** Schematic of transcription cycle stages targeted by triptolide, to inhibit initiation, and α-amanitin, to inhibit elongation, and overview of experimental timeline. **(b)** RPB1 diffusion coefficient distributions in vehicle and triptolide injected embryos in nc13 (left) and nc14 (right). **(c)** RPB1 diffusion coefficient distributions in vehicle and α-amanitin injected embryos in nc13 (left) and nc14 (right). 210,638 trajectories from 9 embryos (270 nuclei) in nc13 and 205,532 trajectories from 10 embryos (264 nuclei) in nc14 were obtained for vehicle injected embryos; 40,775 trajectories from 3 embryos (70 nuclei) in nc13 and 57,003 trajectories from 5 embryos (84 nuclei) in nc14 were obtained for triptolide injected embryos; 37,885 trajectories from 5 embryos (74 nuclei) in nc13 and 55,474 trajectories from 6 embryos (82 nuclei) in nc14 were obtained for α-amanitin injected embryos. **(d)** Comparison of bound (left), intermediate (center), and fast (right) fractions for RBP1 in nc13 and nc14 for all conditions. Data points represent 23 individual fields of view for vehicle-injected embryos in both nc13 and nc14, 9 and 14 individual fields of view for triptolide injected embryos in nc13 and nc14 respectively, and 11 and 16 individual fields of view for α-amanitin injected embryos in nc13 and nc14 respectively with ~8–15 nuclei per field of view. All error bars show standard deviations. Mann-Whitney U-test was performed to determine significance and following p-values were used: *p<0.05, **p<0.01, and ***p<0.001. **(e)** RNAPII bound fraction split into non-specific binding, initiating/paused, and elongating molecules in nc13 and 14.

To assess the relative contributions of molecules engaged in transcription initiation and elongation to the bound fraction of RNAPII, we injected embryos with either triptolide, an inhibitor of transcription initiation^30^, or α-amanitin, an inhibitor of transcription elongation^31^ (Fig. 2a). Using the MS2-MCP reporter system and lattice light-sheet imaging, we determined that injection of either inhibitor during nc12 completely ablates detectable nascent transcription within 5 minutes while still allowing embryos to develop until the mid-point of nc14 after which they fail to gastrulate (Fig. S2.1a–c, Supp. Movies 2–4). Furthermore, we measured the mean nuclear intensity of RNAPII across vehicle, triptolide, and α-amanitin injected embryos in nc13 and nc14 and found no significant differences suggesting that neither injection led to global degradation of RNAPII over the time course of our imaging experiments (Fig. S2.1d). Additionally, the bound, intermediate, and fast fractions of RNAPII do not change between uninjected and vehicle-injected embryos, demonstrating that vehicle injection itself does not significantly alter RNAPII activity (Fig. S2.2).

Inhibiting transcription initiation causes a significant reduction in the bound fraction of RNAPII, with a 2.6-fold decrease in nc13 and a 3.6-fold decrease in nc14 compared to control embryos (Fig. 2b,d and Fig. S2.1e). This decrease in the bound fraction is consistent with triptolide’s mechanism of action, which blocks pre-initiation complex (PIC) formation by binding to the XPB helicase subunit of TFIIH, a key general transcription factor (GTF)^32^. This inhibition reduces the pool of RNAPII available for chromatin engagement. In both nc13 and nc14, 14±5% of molecules remain bound after inhibiting initiation. We interpret this residual bound fraction as non-specific RNAPII-chromatin interactions though we cannot exclude the possibility that some molecules are not evicted by triptolide. In comparison, treatment by α-amanitin decreased the bound fraction by 1.7-fold in nc13 and 2.3-fold in nc14 compared to control embryos (Fig. 2c,e, S2.1e). We reasoned that the residual bound fraction after inhibiting elongation reflects a mixture of paused RNAPII molecules that have initiated transcription and non-specifically interacting molecules. This is consistent with previous studies which suggest that α-amanitin traps RNAPII in an open inactive conformation by interfering with the trigger loop and bridge helix, causing it to cycle between initiation and eviction from chromatin^31,33,34^.

Based on these analyses and our observation that the bound fraction does not change from nc13 to nc14 upon disrupting either elongation or initiation, we decomposed the bound fraction into paused, elongating, and non-specifically interacting molecules (Fig. 2e). This decomposition shows that the elongating fraction of RNAPII increases by ~1.8-fold from nc13 to nc14. Prior work suggests that the properties of RNAPII clusters, including their numbers and lifetimes, depend on the levels of transcriptional activity. This prompted us to next examine our single molecule results in the context of changes in RNAPII clustering during ZGA^13,15,35^.

### Transcription initiation drives cluster formation while elongation destabilizes them

**Fig. 3.**
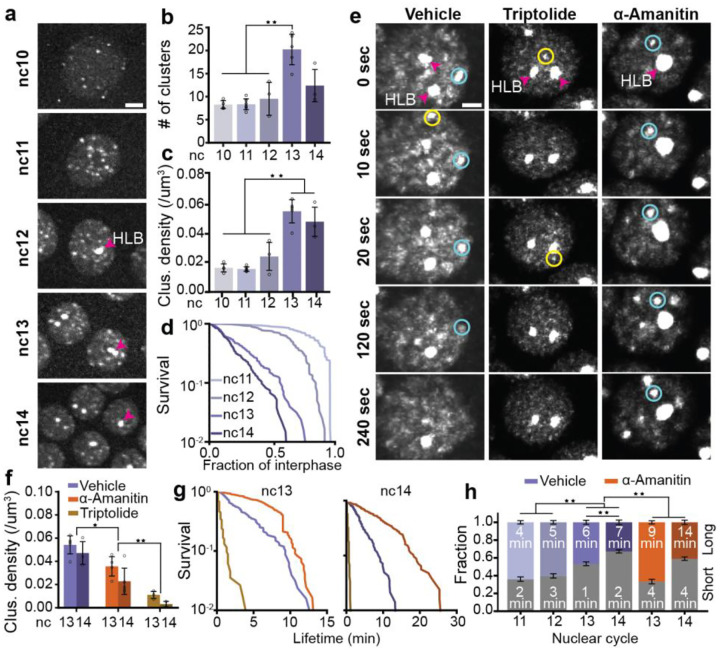
RNAPII cluster kinetics through Zygotic Genome Activation and upon inhibition of transcription. **(a)** Deconvolved maximum-intensity projection images of nuclei from ncs 10–14. Pink arrow indicates a histone locus body (HLB). Scale bar is 6 μm. Quantification of **(b)** number of clusters and **(c)** cluster density (number of clusters per μm^3^ in the nucleus) from nc 10–14. Data points represent individual embryos and error bars show standard deviation. n = 4, 6, 3, 5, and 3 embryos for nc 10, 11, 12, 13, and 14 respectively. **(d)** Survival curves for cluster lifetimes normalized to the duration of interphase from nc11–14. Survival curve for nc10 is not shown because all clusters last the entire duration of interphase. 120 clusters were measured for each nuclear cycle from at least 3 embryos with at least 5 nuclei per embryo. **(e)** Image time series of a nucleus from a vehicle-injected embryo (left), triptolide-injected embryo (middle), and an α-amanitin-injected embryo (right). All image intensities are scaled identically. Yellow and blue circles are examples of short- and long-lived clusters respectively, as determined by fitting the distribution of lifetimes to a two component model. Pink arrow indicates representative HLBs (the two brightest clusters in each nucleus). Scale bar is 2 μm. **(f)** Cluster density in vehicle, triptolide, and α-amanitin injected embryos in nc 13 and 14. Data points are individual embryos. n=5, 3 for the control embryos in nc 13, 14 respectively, n=3 for both triptolide and α-amanitin injected embryos in nc 13,14. Error bars represent standard deviation. **(g)** Cluster survival probability for vehicle, triptolide, and α-amanitin in nc 13 and 14. 120 clusters were measured for each condition and nuclear cycle over at least 3 independent movies and at least 5 nuclei per movie. **(h)** Cluster fractions of short-lived clusters (in grey, bottom) and the long-lived clusters (in color, top) for vehicle and α-amanitin embryos. Text inside each bar shows the mean lifetime of clusters for that condition (nuclear cycle and treatment) and category (short, long) in minutes. Error bars represent standard deviations of each component mean, calculated from the square roots of the covariances provided by the Gaussian Mixture Model fitting. triptolide is not shown due to low cluster counts and no long-lived population. Mann-Whitney U-test was performed to determine significance and following p-values were used: *p<0.05, **p<0.01, and ***p<0.001.

To examine RNAPII clustering during ZGA, we performed volumetric lattice light-sheet imaging in *Drosophila* embryos with endogenous RPB1 tagged with eGFP (Fig. S1.1). We found that RNAPII forms distinct clusters as early as nc10 while the more prominent Histone Locus Bodies (HLBs) first emerge at nc12 (Fig. 3a, Fig. S3.1, and Movie 5). The HLBs are easily identified as the two largest and brightest RNAPII bodies in each nucleus and form exclusively at clusters of replication dependent histone genes which contain ~100 copies of each histone gene^36^. Quantification of the detectable cluster numbers, excluding HLBs, show that the average number of clusters per nucleus is initially stable from nc10–12 at ~8/nucleus, and sharply increases by ~2-fold in nc13. Cluster number then decreases down to ~12/nucleus in nc14 (Fig. 3b, Fig. S3.2). Although the nuclear volume decreases by ~29% from nc 10 to 12, the cluster density (number of clusters per μm^3^) remains relatively stable during this period^37^. However, in nc13, the ~2-fold increase in cluster number combined with a 46% decrease in nuclear volume resulted in a pronounced increase in cluster density. Notably, as the nuclear volume continues to decrease further by ~37% from nc13 to nc14^37^, the cluster density remains relatively constant (Fig. 3c).

Next, we investigated whether changes in cluster number and density were accompanied by changes in cluster lifetimes. Since interphase times increase significantly during *Drosophila* embryogenesis from nc11 (~6–7 minutes) to nc14 (~20 minutes prior to cellularization), we normalized individual cluster lifetimes to their respective interphase lengths^38^ (Fig. S3.3a). While the majority of the clusters in the early cycles (nc11–12) last almost the entire interphase, we found that normalized lifetimes are strikingly shortened as ZGA progresses (Fig. 3d). The distribution of cluster lifetimes are well described by a short and long-lived population (Fig. S3.3b,c, Movie 8). The fraction of long-lived clusters decreases from 64±2% in nc11 down to only 33±3% in nc14. The data suggest that cluster lifetime may not be driven by cycle length but rather possibly the transcriptional cycle. To test this, we analyzed cluster lifetimes upon inhibition of elongation or initiation.

Upon inhibiting transcription initiation, we found that cluster formation is almost completely abrogated with the exception of HLBs (Fig. 3e,f, Movies 7,10). In contrast, inhibition of elongation leads to a moderate decrease in cluster density (Fig. 3e,f, Movies 6,9) but a sharp increase in both the cluster lifetime and fraction of clusters in the long-lived population. In control embryos, the mean long-lived cluster lifetime is 6±2 minutes in both nc13 and 7±1 minutes in nc14. However, in α-amanitin-injected embryos, the mean long-lived cluster lifetime increases to 9±2 minutes in nc13 and 14±3 minutes in nc14, reflecting a 1.5-fold increase in nc13 and a 2-fold increase in nc14 compared to the cluster lifetimes measured in control embryos (Fig. 3g, Fig. S3.3). Furthermore, 67±2% and 41±2% of the clusters are long-lived in the α-amanitin-injected embryos in nc13 and nc14 respectively, reflecting a 1.4-fold and 1.2-fold increase over the corresponding fractions in control embryos (Fig. 3h).

Together, these data show that transcription initiation is necessary for RNAPII cluster formation while elongation de-stabilizes clusters, perhaps due to a higher turnover of RNAPII at genes. To further understand what regulates clustering kinetics, we next quantified the molecular kinetics of RNAPII within clusters.

### RNAPII clusters are composed of elongating and kinetically trapped molecules after ZGA

**Fig. 4.**
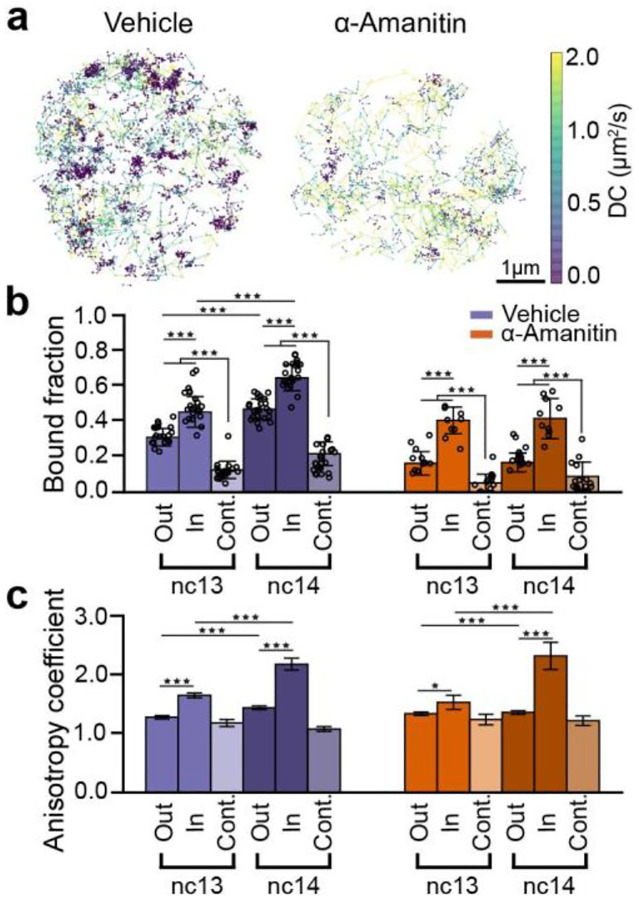
Single molecule kinetics of RNAPII inside clusters during ZGA. **(a)** Representative nuclei showing single molecule tracks for RPB1-mEos3.2 color coded by the diffusion coefficient (colorbar to the right) for vehicle and α-amanitin injected embryos. Tracks are shown from a 60 sec total imaging window with an exposure time of 10 msec. **(b)** Bound fraction of RPB1 inside clusters, outside clusters and in control spots for vehicle and α-amanitin injected embryos in nc13 and nc14. Data points represent 23 individual fields of view for control embryos in both nc13 and nc14, and 11 and 12 individual fields of view for α-amanitin injected embryos in nc13 and nc14 respectively (~8–15 nuclei per field of view). Error bars show standard deviations. *p<0.05, **p<0.01, and ***p<0.001 **(c)** Anisotropy coefficient of the trajectories outside clusters, inside clusters and inside control spots for the vehicle and α-amanitin-injected embryos. Number of angles in vehicle-injected embryos are 13,107, 3,070, and 5,754 for outside clusters, inside clusters and in control spots respectively in nc13 and 10,540, 2,057, and 6,104 for the same categories in nc14. Number of angles in the α-amanitin-injected embryos are 2,664, 995 and 2,189 for outside clusters, inside clusters, and in control spots respectively in nc13 and 4,807, 1125, and 2,894 for the same categories in nc14. Error bars represent standard deviation from bootstrapping analysis. Mann-Whitney U-test was performed to determine significance and following p-values were used: *p<0.05, **p<0.01, and ***p<0.001

Consistent with our volumetric imaging data, our single molecule tracks exhibit clustering in vehicle-injected embryos which is reduced in α-amanitin injected embryos (Fig. 4a). We identified clusters based on the local density of trajectories and then filtered these clusters based on size and density to exclude likely HLBs and random accumulations (Fig. S4.1). By comparing RNAPII kinetics inside clusters versus the rest of the nucleoplasm (outside clusters), we found a ~1.4 fold enrichment of bound RNAPII trajectories inside clusters in both nc13 and nc14 in vehicle-injected embryos and ~2.5-fold enrichment of bound tracks inside clusters in both nc13 and nc14 in α-amanitin injected embryos (Fig. 4b). To ensure that the observed enrichment inside clusters is not an artifact resulting from performing analysis within restricted regions of a nucleus, we quantified the bound fraction in control regions of the same size as the clusters but located randomly within the nucleus and not overlapping with clusters. In vehicle-injected embryos, the bound fraction inside clusters was 3.5-fold and 3.0-fold higher than in control spots in nc13 and nc14 respectively (Fig. 4b). The bound fraction within clusters does not increase from nc13 to nc14 in α-amanitin injected embryos, similar to the global population (Fig. 2). In sharp contrast, in vehicle injected embryos, we found a 1.4-fold increase in the bound population. A decomposition of this bound population as done above (Fig. 2e) leads to an estimate of a nearly 6-fold increase in the elongating population of RNAPII molecules within clusters from nc13 to nc14 (Fig. 4c).

To quantify how the kinetics of molecules in the non-chromatin bound (intermediate and free) populations are altered within clusters, we calculated the distribution of angles between three consecutive displacements and quantified changes in the anisotropy coefficient of tracks within and outside clusters^39^ (Fig. S4.2). We found that the anisotropy coefficient inside clusters remained consistently higher than both outside clusters and control spots in nc13 and nc14 in both vehicle and α-amanitin injected embryos (Fig. 4c). The increased anisotropy in clusters suggests that molecules within them are exhibiting compact exploration kinetics and are likely kinetically confined through frequent rebinding or through an increase in local protein-protein interactions. Furthermore, in vehicle injected embryos, the anisotropy coefficient inside clusters exhibits a ~1.3-fold increase from nc13 to nc14 (Fig. 4c). Overall, these analyses show there is an increase in the elongating fraction of RNAPII within clusters concurrent with an increase in the kinetic confinement of unbound RNAPII molecules during ZGA.

### A single RNAPII cluster persistently associates with a site of active transcription

**Fig. 5.**
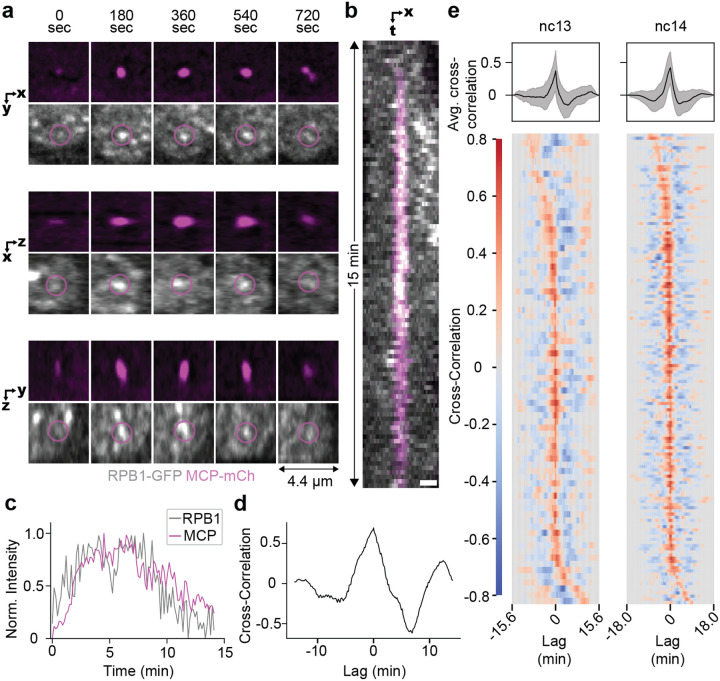
RNAPII clusters “burst” concomitantly with a transcription burst. **(a)** Orthogonal view images of a nucleus in nc14 with RPB1 (grey) and MCP-mCherry (pink) labelling active transcription at a *hb*-MS2 reporter during a ~15 min transcriptional burst. Cytoplasmic MCP signal is present as it is expressed without a nuclear localization signal. **(b)** Kymographs for the same nucleus calculated as max-projection over a 1.2 μm y-slice. Scale bar is 1 μm. **(c)** Normalized intensity of RNAPII and MCP in a 1.2μm diameter circle around the *hb*-MS2 spot of the same nucleus shown in a. **(d)** Cross-correlation of the RNAPII and MCP intensity traces from (c). **(e)** Average (top) and individual (bottom) cross-correlation of nuclei in nc13 (n = 76) and nc14 (n = 149) from 4 biological replicates. Gray shaded area in line plots represent standard deviation.

To determine the behavior of RNAPII clusters at a specific site of active transcription, we examined clustering in the context of nascent transcription using the MS2-MCP system. We visualized transcription at a reporter for the gene hunchback (*hb*), which is active in the anterior segments of the *Drosophila* embryo during nc13 and 14. We performed two-color lattice light-sheet imaging, sequentially acquiring a volume in each channel every 10 seconds. We observed that at least one RNAPII cluster was consistently associated with the *hb* locus throughout a transcription burst (Fig. 5a,b, Fig. S5.1–2). The intensity of RNAPII at the *hb* locus is highly correlated with the intensity of the nascent transcript during a transcriptional burst (Figs. 5c–d). Cross-correlation analysis of nascent transcript and RNAPII intensity traces consistently exhibits a strong peak at zero lag times, indicating a consistent temporal synchronization (Fig. 5e). Thus, a RNAPII cluster accumulates at the *hb* locus at the start of a transcription burst, and the concentration of RNAPII within those clusters (as implied by the cluster intensity) is correlated with the level of transcriptional activity. As the RNAPII clusters become less intense, the transcriptional levels also drop, and eventually the cluster dissipates. Together with our single-molecule data, the high correlation between RNAPII intensity and transcription levels at a single gene suggest that, after ZGA, clusters represent sites of transcription elongation at an active gene.

## Discussion

**Fig. 6.**
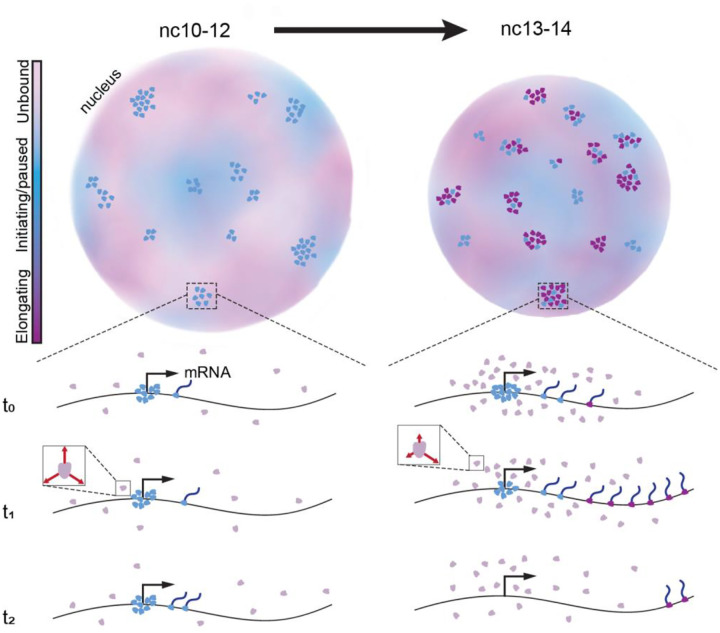
Functional transformation of RNAPII clusters during ZGA. In earlier nuclear cycles (nc10–12), RNAPII clusters are primarily comprised of initiating and paused RNAPII molecules. As transcription increases in later cycles (nc13–14) clusters are associated with elongation. When transcription activity peaks in nc14 molecules within clusters exhibit an increased kinetic confinement as indicated by red arrows in the inset.

Here we show that during the widespread increase in transcriptional activity during ZGA, RNAPII clusters transform from collections of largely paused and initiating molecules, to primarily elongating molecules. Our data may help reconcile contradictory reports in the literature on RNAPII cluster formation as either occurring only at pre-elongation/initiation steps^12,13,40^ or upon pause release/elongation^14^. Our data instead suggest that RNAPII clusters play a role in priming transcriptional activity prior to ZGA, and largely represent regions of high transcriptional activity after ZGA (Fig. 6). Furthermore, through perturbations to the transcription cycle and live imaging of transcription, we conclude that RNAPII cluster formation is dependent on pre-elongation stages and once polymerases within clusters are released into elongation, the clusters persist at an active locus during the duration of a transcription burst.

In the nuclear cycles prior to the major wave of ZGA (nc10–12), RNAPII clusters exhibit lifetimes comparable to interphase durations (Fig. 3d) consistent with low turnover of molecules and high propensity for aborted transcription^20^. This early-stage clustering may poise developmental genes for activation as has been described in the context of dorso-ventral patterning during ZGA^41^ and also during later developmental stages coinciding with tissue specification^42^. Starting in nc13, there is a sudden reduction in the fraction of long-lived clusters and increase in the nuclear density of clusters (Fig. 3c). Despite the decrease in cluster lifetimes, we observe an increase in the anisotropy of non-bound RNAPII within clusters during nc14 which suggests a kinetic confinement of molecules near active transcription sites (Fig. 4). This confinement could be a result of the well documented role of protein-protein interactions in mediating RNAPII clustering through its C-terminal domain^43–45^. The shift in cluster lifetimes and densities likely reflects the release of a greater fraction of RNAPII molecules from paused states into elongation as indicated by a reversion of lifetimes and densities upon elongation inhibition (Fig. 3f–h). An increase in RNAPII cluster lifetimes upon elongation inhibition via DRB or flavopiridol has also been observed in cell culture studies^13^. The dissolution of transcriptional condensates upon an increase in transcriptional activity has also been reported and is proposed to arise from changes in electrostatic forces from local RNA concentrations^19^. The high correlation of local RNAPII intensity and transcriptional output we measure (Fig. 5) suggests that the reduction of cluster lifetime may be simply related to an increase in the number of RNAPII molecules being released into the gene body rather than higher order mechanisms. Furthermore, the intensity correlation we observe is consistent with previous interpretations of the shape of a transcriptional burst representing the kinetics of the number of RNAPII molecules being loaded onto and elongating at a gene body^46,47^.

The strong correlation between mRNA production at a single active gene and RNAPII cluster intensity suggests that each cluster could be associated with a single gene. However, despite the increase in transcriptional activity in nc14, the nuclear cluster density does not increase compared to nc13 (Fig. 3c) and the number of countable clusters per nucleus is far lower than the number of genes being transcribed. This discrepancy could stem from several factors but the two most likely are: (1) most genes don’t recruit enough polymerases at a time to form clusters that arise above the background nuclear signal in live imaging, (2) clusters contain multiple co-bursting genes^48^ within or at the boundaries of topologically associated domains (TAD). The latter point is unlikely given the number of active genes (~3500) compared to number of detectable clusters, but is supported by Hi-C studies in *Drosophila* embryos which show that RNAPII plays a crucial role in establishing new TAD boundaries and mediating inter-TAD compaction during development, which may influence cluster formation and function^49^.

Our observations align well with an individualistic model of gene regulation by clusters^11^, that is a single RNAPII cluster per gene. Furthermore, our observation of a stable association of an active gene with a cluster contrasts with the observation of larger RNAPII condensates engaging in transient “kiss-and-run” interactions with genes to enhance transcriptional bursting^17^. These larger stable RNAPII condensates are potentially equivalent to histone locus bodies (HLBs) as implicated by recent proteomics profiling^50^. HLBs in our own and others^11^ experiments persist even after inhibition of elongation, and may enhance transcription differentially than the majority of clusters that we focus on in this work. Our results also contrast with previous observations in *Drosophila* embryos that suggest RNAPII cluster intensities peak before transcription and that clusters diminish well before the end of a transcription burst suggesting that only a minority of RNAPII molecules recruited to the initial cluster contribute to productive elongation^18^. This discrepancy may potentially arise from methodological differences as in this previous study^18^ the aggregate intensity of all RNAPII clusters in the nucleus was analyzed and we directly measured the intensity of RNAPII clusters at an actively transcribing gene.

Overall, our data suggest that RNAPII clusters are not limited to a single functional state but are functionally labile. In earlier developmental cycles, they are composed primarily of initiating and paused molecules, transitioning in later cycles to include elongating states. Furthermore, our results highlight that RNAPII clusters are stably associated with transcription sites. Future studies should explore the molecular triggers for these functional transitions, the initial drivers of RNAPII cluster formation in relation to other core components of the pre-initiation complex, and the role of changes in local chromatin density and topology in modulating cluster lifetimes.

## Materials and Methods

### Western Blots

Embryos were collected 30 minutes after egg-laying and incubated for 75 minutes at 25°C. They were then dechorionated with 50% bleach and staged in Halocarbon oil 27. Approximately 90–100 embryos were collected in 1x PBS + PIC in 4x Laemmli SDS sample buffer with 5% BME. The embryos were homogenized on ice using disposable pestles, heated to 95°C for 5 minutes, and allowed to cool to room temperature. Samples were then centrifuged at 4°C for 10 minutes at 16,000 RPM. The supernatant was transferred to new 1.5 ml tubes and stored at −20°C until needed. Protein samples were split into two equal volumes and run on an 8% acrylamide gel at 150 volts for 5 hours for Phospho-RPB1 CTD (Ser5) protein separation and for 1 hour for beta-tubulin separation. Proteins were transferred onto nitrocellulose membranes overnight at 20 mA overnight at 4°C. All membranes were blocked for 30 minutes with 3% milk in 1X TBST (1X TBS, 0.2% Tween) at room temperature. The blots were then incubated overnight at 4°C with either rabbit anti-Ser5 at 1:250 (Abcam; ab5131) or rabbit anti-beta-tubulin (Abcam; ab179513) at a 1:500 dilution. An anti-rabbit HRP antibody was used as a secondary at 1:10,000 for 1 hour at room temperature. Blots were treated with ECL substrate and visualized using the BioRad ChemiDoc system.

### Embryo collection and mounting for live-imaging

Embryos were incubated at 25°C for 45 minutes and transferred from an apple juice-agar collection plate to a cell strainer immersed in a petri dish of water. Embryos were dechorionated by incubating in 6% sodium hypochlorite for 51 seconds, then immediately rinsed with distilled water. Embryos were then transferred and positioned in rows on an agar pad using a fine paintbrush and dissecting needle under a dissection microscope. A 25 mm glass coverslip was coated with 8 μl of double-sided Scotch tape dissolved in heptane to make it adhesive. Once the heptane was fully evaporated, embryos were transferred to the coverslip by gently tapping. A drop of distilled water was added to hydrate the embryos on the coverslip.

### Embryo microinjection

Embryos were collected, mounted, and imaged with a lattice light-sheet microscope to determine their developmental stage. Embryos in nc12 were identified for injection and the sample was transferred for injection. Distilled water was removed from the coverslip using a Kimwipe, and a drop of Halocarbon oil 27 (Sigma Aldrich) was added to the embryos. Microinjections were conducted using an Eppendorf Femtojet mounted on a Leica DMi8 Microscope. A Sutter P-87 needle puller was programmed to pull “medium taper” needles at Heat = 915°C, Pull = 80, Velocity = 28 and Time = 250 using thin-wall borosilicate capillaries (WPI TW100F-4). Pulled needles were calibrated for injection by ensuring they consecutively produced 10 bubbles of similar diameters in halocarbon oil. Pulled microinjection needles were loaded with 3.5 μL of either α-amanitin, triptolide or the vehicle control to be injected. Step injection was conducted at 0.1 sec, with a compensatory pressure of 15kPa. Post injection, halocarbon oil was gently washed off the embryos with distilled water and remounted on the lattice light-sheet microscope for imaging of the injected embryos. α-amanitin (Sigma Aldrich) was dissolved in water at a concentration of 0.5 mg/ml. Triptolide (MedChem Express) was dissolved in DMSO at 1 mg/ml and diluted with water at a 1:1 ratio. DMSO was diluted in water in a 1:1 ratio for the negative control experiment. Each embryo was injected with approximately 0.2 nL of the solution^49^. All embryos were mounted for imaging within five minutes of microinjection.

### Microinjection Survival Assay

Mounted His2B-eGFP embryos were imaged on a Leica DMi8 Microscope and injected in different nuclear cycles to determine survivability. Nuclear cycles were determined by bulk histone H2B-GFP markers in the nuclei. Microinjections were conducted and then development to gastrulation was noted. Based on the survival assay, it was determined that embryos that were injected with drugs in nc12 or later showed normal development until the mid-point of nc14 as evidenced by regular division cycles and normal morphology. Embryos injected with drugs prior to nc12 showed significant developmental defects within ~10 minutes, well before nc14. Embryos that were injected with the vehicle did not show any defects and proceeded to gastrulation.

### Light-sheet microscope optical paths and configuration

The lattice light-sheet microscope^52^ used in this work is a modified, home-built implementation based on the adaptive optics-equipped lattice light-sheet system developed by the Betzig lab at HHMI Janelia Research Campus and UC Berkeley^53^. For experiments in this work the following laser lines were used: 405 nm (Coherent), 488 nm (MPB Communications Inc.) and 561 nm (MPB Communications Inc.). Briefly: the laser lines were expanded to a diameter of 2 mm and combined using a series of dichroic mirrors, passed through a Half-wave plate (Bolder Vision Optik) to adjust polarization and relayed into an acousto-optic tunable filter (Quanta-Tech, AA Opto Electronic) to select wavelength and modulate power. The output from the AOTF was then either sent to an optical path to generate a lattice light-sheet excitation pattern or a multi-gaussian beam excitation pattern. For lattice light-sheet generation the collimated laser beams were expanded along a single dimension using a Powell Lens (Laserline Optics Canada) and then the width of the expanded beam was adjusted and re-collimated using a pair of cylindrical lenses (25 mm diameter; Thorlabs). This stripe of collimated light was relayed onto a grayscale Spatial Light Modulator (SLM; Meadowlark Optics, AVR Optics) after passing through a second Half-wave plate. The SLM is conjugate to the sample-plane of the microscope. The diffracted light from the SLM was passed through a lens to project its Fourier transform onto a custom built annular mask to select the minimum and maximum numerical aperture of the light-sheet and block unwanted diffraction orders from the SLM. The annular mask is positioned in the pupil plane of the excitation light path. The annular mask plane was demagnified and projected onto a resonant galvanometer (Cambridge Technology, Novanta Photonics) conjugate to the sample plane. The resonant galvanometer was used to mitigate shadowing artifacts and other inhomogeneities in the light-sheet by introducing a slight wobble in the excitation angle. The light was then projected onto a pair of galvanometer scanning mirrors (Cambridge Technology, Novanta Photonics) (conjugated to the pupil plane) for scanning the light-sheet along x and z optical axes in the excitation coordinate plane. Finally, an excitation objective (Thorlabs, TL20X-MPL) was used to focus the light-sheet onto the sample. The emitted fluorescence was collected by a detection objective oriented orthogonally to the excitation objective (Zeiss, 20×, 1.0 NA), and projected onto a Deformable mirror (ALPAO) positioned in a pupil of the detection path. The light was then split using a dichroic beam splitter (Semrock Di03-R561-t3-25×36) and imaged onto two sCMOS detectors (Hamamatsu ORCA Fusion). The first camera had a green emission filter (Semrock FF03-525/50-25) and a notch filter (Chroma ZET488NF) to reject laser light and the second camera had a red emission filter (Semrock FF01-593/46-25) and a notch filter (Chroma ZET561NF) to reject laser light. Optical aberrations in the detection path were corrected by adjusting the deformable mirror (Alpao), as previously described^53^.

### Single-molecule imaging and tracking using light-sheet microscopy

For single-molecule imaging a Gaussian light sheet was used (see the section on “Light-sheet microscope optical paths and configuration” for more details). Briefly, after passing through the AOTF, the beam was expanded using a Powell lens following which it was relayed onto a custom mask for filtering. The filtered sheet was then projected onto a resonant galvanometer (Cambridge Technology, Novanta Photonics). Finally, an excitation objective (Thorlabs, TL20X-MPL) is used to focus the light-sheet onto the sample. The detection path for the emitted light is the same as described above for the lattice light-sheet configuration. For this light path, the SLM was bypassed to maximize laser power at the excitation objective.

For all the single-molecule imaging, the 405 nm laser line was kept on constantly during the acquisition period for photoswitching and the 561 nm laser line was used for excitation. Data was acquired at 10 msec exposure time. Excitation laser power was empirically optimized to maximize contrast for single-molecule tracking while minimizing photobleaching. The power of the photoswitching laser was also optimized to maintain a low enough detection density for tracking. The excitation laser power used was 13 mW and switching laser power used was 1 μW as measured at the back focal plane of the excitation objective. A total of 8,000 frames were acquired corresponding to 80 sec of total imaging time. The acquisition length was optimized such that multiple fields of views could be imaged within each short interphase time while also capturing a sufficient number of trajectories at each position. To optimally position the embryo in the light sheet and to keep track of cell-cycle phase, and nuclear cycle, His2B-eGFP was used.

### Volumetric imaging using lattice light-sheet microscopy

Live volumetric imaging of the RPB1-eGFP line was done by using a multi-bessel lattice sheet with maximum numerical aperture to minimum numerical aperture ratio of 0.4/0.3. A 488 nm laser was used for the volumetric imaging with laser power of 0.512 μW and exposure time of 60 msec. For each time point, 64 slices were imaged at a thickness of 300 nm per slice for a total depth of 18.9 μm. Each volume was acquired every 10 sec. For sequential two-color imaging of the RPB1-eGFP with the hb-MS2-MCP-mCherry, the 488 nm laser was used for exciting eGFP while the 561 nm was used for exciting the mCherry. Laser powers of 0.512 μW and 1.40 μW were used for the 488 nm and 561 nm wavelengths respectively at an exposure time of 40 msec. The two channels were acquired sequentially for a total volume of 18.9 μm. For each channel, 64 slices were imaged at a thickness of 300 nm per slice. Each volume was acquired every 9 seconds during acquisition.

### Processing of single-molecule tracking data for quantifying diffusion kinetics

To process the SMT data, we first removed the initial ~2,000 frames affected by fluorophore bleaching. Maximum projections were generated to obtain nuclear masks. Localization and tracking of the single molecule trajectories was performed using the open source software package quot (https://github.com/alecheckert/quot). For the 10 msec data the following key parameters on quot were used: (i) for detection - method = “identity”, Spot detection settings = “llr”, k = 1.3, w = 15, t = 21, (ii) for localization - method = “ls_int_gaussian”, window size = 9, sigma = 1.0, ridge = 0.0001, maximum iterations = 10, damp = 0.3, camera gain = 1, camera background = 108 (iii) for tracking - method = “euclidean”, pixel size (in μm) = 0.108, search radius = 0.7 μm, maximum blinking = 0 frames. Once we obtained our list of tracked molecules from quot, we filtered them using the nuclear mask as described earlier to ensure we analyzed only trajectories that were occurring inside the nucleus.

### Analysis of single-molecule tracking data

To analyze the 10 msec single molecule trajectories to infer the diffusion kinetics, we used a variational Bayesian method called State Array based Single Particle Tracking^28^. This method does not a priori assume a specific number of diffusive states. Instead, it processes all the recorded trajectories and selects a model that comprehensively describes the trajectories with the minimum combination of state parameters (diffusion coefficient and localization error) as possible. Consequently, it produces average posterior state occupancies for a state array evaluated on the experimental trajectories. For the model selection, we set a range of 100 diffusion coefficients extending from 0.001 μm^2^/s to 100 μm^2^/s representing a range of biologically plausible diffusion coefficients. This package is publicly available at https://saspt.readthedocs.io/en/latest/. For our analysis, we used the following specific parameters in the saSPT program:

Likelihood type: RBME
Pixel size: 0.108 (1 pixel = 0.108 μm)
Frame interval: 0.0125433 msec
Concentration parameters: 1
Max iterations: 200
Split Size: maximum trajectory length
Sample Size: number of trajectories

Using saSPT, we were able to generate the diffusion coefficient occupancy plots for each protein category. We identified the local maxima and minima by taking the derivative of the occupancy plots and assigned the different kinetic states as follows: (i) the first state extends from the lowest DC value of 0.001 μm^2^/s to the first local minima detected; (ii) the last state extends from the last local minima detected to the highest DC value of 100 μm^2^/s; (iii) all other states extend between consecutive minima. We used the minima obtained from the vehicle injection in each nuclear cycle to assign the states for the drug conditions in that corresponding nuclear cycle.

Furthermore, in order to assign individual diffusion coefficients to each trajectory for downstream analysis, we first weighted the range of diffusion coefficients by their corresponding occupancies for each trajectory. We calculated the geometric mean of these weighted coefficients which was then assigned to each trajectory. To estimate the localization error in our single-molecule tracking data, we analyzed the subset of H2B-mEos3.2 trajectories with the lowest diffusion coefficients. We calculated the mean of their consecutive displacements over time and determined that the localization error was 30 nm.

For the anisotropy analysis, we first filtered the data sets to remove all the bound trajectories since they can bias the angle distribution. We then calculated the angle between all consecutive jumps (Fig. S4.2b,c) while excluding all jumps that were less than 0.2 μm based on the jump distribution of His2B (Fig. S4.2a). We then calculated the fold-anisotropy metric as the probability of observing a backward jump (with an angle between jumps in the range [180° − 30°,180° + 30°]), divided by the probability of observing a forward jump (with an angle between jumps in the range [0 − 30,0 + 30]).

To determine the clustering behavior from the single molecule trajectories, the average position of the trajectories were first calculated by considering the mean x and y locations. Average trajectory positions were considered because we wanted to quantify the diffusion kinetics inside the clusters and hence needed to assign diffusion coefficients to individual trajectories. We then used density-based spatial clustering of applications with noise (DBSCAN) to determine the location of the clusters and the trajectories that comprised the cluster. For the DBSCAN analysis, we specified that there had to be a minimum of 15 points (“min. samples”) to define a cluster and the maximum distance between any two points in the cluster was 0.2 μm (“max. distance”). These values were selected by initially plotting the number of clusters as a function of the max. distance to determine where the plot showed an elbow, i.e. after what threshold value of max distance did the number of clusters did not change significantly, a common analysis technique used in DBSCAN to determine the appropriate parameters (Fig. S4.1b). The parameters chosen were further validated by qualitatively comparing the DBSCAN predictions to the raw spatial maps of the single molecule trajectories. These values were kept constant between all the protein categories and nuclear cycles.

For any nucleus in which clusters were identified by DBSCAN, we also defined ten control spots per cluster identified. These control spots were assigned such that they satisfied the following four criteria: (i) they did not overlap with any of the DBSCAN identified clusters in that nucleus, (ii) they did not overlap with each other and (iii) they were of the same size as the DBSCAN identified clusters and (iv) they were within the nuclear mask. The kinetics inside these assigned spots were used as a negative control against the measurement of bound fraction and kinetics inside the DBSCAN identified clusters.

Single molecule trajectories were taken from a minimum of 3 independent embryos for all the conditions and nuclear cycles analyzed. Detailed trajectory number and the number of fields of views from which the trajectories were compiled can be found in each figure caption. The diffusion coefficient occupancy plots for each condition and cycle was obtained after concatenating the data sets from all the movies acquired. The standard deviation of the bound fractions was calculated across the individual fields of views. The standard deviation for the fold-anisotropy was calculated by bootstrapping 50% of the data 20 individual times. In order to calculate statistical significance where relevant, we used the Mann-Whitney U-test since our data was not normally distributed as confirmed by the Kolmogorov-Smirnov test.

### Analysis of volumetric imaging data to quantify cluster properties

Volumetric imaging data were pre-processed using GPU-accelerated 3D image deconvolution with CUDA^54^ before downstream analysis. All datasets were deconvolved using input PSF taken by bead images on the LLSM and using ten iterations for Richardson-Lucy based deconvolution. We used a custom Imaris converter leveraging fast [Tiff or Zarr] file readers to generate Imaris files for data visualization and rendering^55^. All volumetric images shown in the figures of this manuscript are deconvolved images. After deconvolution, images were subjected to nuclear segmentation. To segment nuclei in our dataset, we created a custom model using Cellpose 2.0^56^. Ground truth data was generated using a mixture of micro-Sam^57^, a Napari plugin for segment anything^57^ and manual correction on wildtype RPB1-eGFP images. This dataset was then used to train Cellpose 2.0. The resulting model was then used to segment all slices of each acquired dataset individually. A custom post-processing pipeline was then utilized in order to stitch the individual slices back together and to interpolate any slices of called objects that were missed in segmentation. We then implemented a nearest neighbor algorithm to track nuclei over the course of interphase in each nuclear cycle.

To quantify cluster properties, we modified a custom analysis pipeline we previously used^27^. In this pipeline, nuclei were first normalized to their mean intensity to assess local enrichments of TFs above nuclear background. This pipeline segments clusters by using a median filter to remove noise followed by image erosion and reconstruction. The reconstructed image was subtracted from the median filtered image to first create a binary mask of high density regions. Then, we called local maxima peaks to be used as markers for watershed segmentation in order to separate clusters that might be fused together in the binary mask. We then used region props to quantify different properties of the clusters such as integrated intensity, mean intensity, and size. This pipeline indiscriminately segments out all clusters including the larger histone locus bodies (HLBs).

To distinguish smaller clusters from the larger clusters surrounding HLBs, we segmented HLB clusters by using a difference of gaussians (sigma high = 5, sigma low = 1) followed by a percentile threshold set by visual inspection (99.95%). Knowing that there are only two or less HLBs in a single nucleus in any given frame, we identified and removed the segmented clusters that overlapped with the segmented HLBs and had the highest measured cluster enrichment. Thereby, resulting in separate masks for RPBI-GFP clusters and HLBs. We then filtered these clusters (without HLBs) by their mean enrichment. Using empirical testing, we found that an enrichment of about 1.65 was appropriate for segmenting the RPB1 clusters without picking up on background noise. We used this cut off as a means to clear any cluster that might have a minimal and non-significant enrichment compared to the nuclear mean intensity. To calculate cluster density we divided the total number of clusters in each nucleus by the corresponding nuclear volume.

### Analysis of MS2-RNAPII interactions

The center coordinates of each MS2 spot was identified by first segmenting each spot. A difference of gaussians (sigmas = 1.5 and 6) followed by a percentile threshold (each threshold manually verified by visualizing multiple frames throughout the time course) was used. Small objects below 6 pixels were removed as noise. Cytoplasmic objects were removed if they were outside the nuclear labels. To account for multiple MS2 spots per nucleus (due to replication), the highest intensity spot was selected for tracking across frames. From this segmentation, the MS2 spot center was then calculated by using the segmented object’s weighted centroid position. These coordinates were used to generate tiff stacks that were a 5-slice max-projection in z around the z-slice with the brightest MS2 spot at each time point. The coordinates were manually edited to correct any errors in spot tracking. RNAPII and MCP intensity traces were calculated by drawing a 11 pixel (1.2 μm) diameter circle around the MS2 spot at each time point. The MS2 spot was taken as a 2×2 pixel square with the highest MCP intensity in each max-projected z-slice. Full, discrete, linear cross-correlations were computed in Python by centering the intensity traces and using the ‘correlate’ function from the scipy.signal package, with the ‘full’ mode and ‘direct’ method specified as arguments. These were normalized by dividing by the product of the signal standard deviations. The value of these direct correlation at lag = 0 is equivalent to the Pearson correlation coefficient.

Kymographs were generated from these tiff stacks in ImageJ. A 1.2 × 4.4 μm (11 × 41 pixel) slice around the MS2 spot was selected and a kymograph was made for each row. The 11 resulting kymographs were then max-projected to create the final max-projected kymograph.

### Analysis of cluster lifetimes

Cluster lifetimes were manually analyzed using FIJI. Deconvolved TIFF hyperstacks for individual nuclei were loaded on FIJI. Once a cluster was identified, it was tracked till it disappeared. The following criteria was used for tracking: a cluster was tracked as long as it remained within 600 nm (2 z slices at 300 nm per slice) either above or below the z position in the current frame. The start position, start time, end position, end time and total length of the movie were all recorded for each cluster. The normalized lifetime was calculated as the ratio of the total length of the cluster to the total length of the corresponding movie. A minimum of 5 nuclei were stochastically selected per embryo and a maximum of 8 clusters were sampled per nucleus. HLBs were excluded from this analysis except in nc11 where there are no clear, distinguishable HLBs.

## Supplementary Material

Supplement 1 **Fig. S1.1. Constructs used for imaging. (a)** Both constructs were generated using the CRISPR-Cas9 system. Each construct includes a Flag-tag at the N-terminus of the fluorescent protein (eGFP or mEos3.2) and is linked to RPB1 via a long linker. **(b)** Western blots show that eGFP and mEos3.2-tagged RPB1 have similar expression levels to wild-type RPB1, as confirmed by tubulin loading control. The double bands in RPB1 blot correspond to previously reported phosphorylated and unphosphorylated states of the RPB1 C-terminal domain^58^.
**Fig. S1.2: Single molecule tracking quality control. (a)** Representative traces showing the number of detections per frame per nucleus in nc13 (top) and nc14 (bottom). **(b)** Analysis pipeline showcasing how the diffusion coefficient spectrum is divided into different kinetic states based on the local minima values. Local minima are represented by blue dots. The different kinetic states are shown as different shaded regions. **(c)** Heatmaps of the probability distributions of diffusion coefficients obtained for each movie (one field of view/row; 8–15 nuclei per field of view) for nc13 (top) and nc14 (bottom).
**Fig. S2.1: Validation of the microinjection process. (a)** Schematic showing an overview of the different stages of the microinjection process. **(b)** Quantification of the microinjection survival assay for vehicle injections (left), triptolide injections (center) and α-amanitin injections (right). **(c)** Representative images of embryos prior to injection showing hunchback MS2 signal (example spots indicated by pink arrows). Injection with either triptolide or α-amanitin causes the MS2 spots to disappear but after injecting with the vehicle the MS2 spots remain. Late in nc14 (~45 minutes into nc14) the embryo injected with the vehicle continues to develop normally while both of the drug injected embryos show major developmental defects validating the injection process. **(d)** Raw nuclear mean intensity of RNAPII. Data points are individual embryos. For vehicle-injected embryos, n=5 in nuclear cycle 13 and n=3 in nuclear cycle 14. For triptolide- and α-amanitin-injected embryos, n=3 in each nuclear cycle. Error bars represent standard deviation. **(e)** Heatmaps of the probability distributions of diffusion coefficients obtained for the vehicle injection, triptolide injection and α-amanitin injection in nc13 (left column) and nc14 (right column). Each row represents one movie.
**Fig. S2.2: Comparison of vehicle-injected embryos and uninjected embryos shows no difference in the SMT kinetics. (a)** Diffusion coefficient distributions for the uninjected embryo compared to the vehicle embryo in nc13 (top) and nc14 (bottom). A total of 210,638 trajectories across 9 independent embryos and 205,532 trajectories across 10 independent embryos were obtained for nc13 and nc14 respectively for the vehicle case. A total of 203,697 trajectories across 10 independent embryos and 254,957 trajectories across 10 independent embryos were obtained for nc13 and nc14 respectively for the uninjected case. **(b)** Bound, intermediate, and fast fractions for both the uninjected and vehicle cases in nc13 (top) and nc14 (bottom) are shown. Individual data points represent individual movies and error bars represent standard deviation.
**Fig. S3.1: RNAPII clusters are visible from nc10 to nc14.** Time-lapse snapshots showing five consecutive imaging time points (rows) during interphase for each nuclear cycle from nc10 to nc14 (columns). Image contrasts are all scaled equally. Images are maximum intensity projections of deconvolved images over 16.8 μm. Scale bar is 1 μm.
**Fig. S3.2: Characterization of RNAPII clusters. (a)** Volumes of a layer of nuclei on the *Drosophila* embryo surface are acquired during imaging and deconvolved. Scale bar is 12 μm. **(b)** Nuclei are segmented in 3D using a trained machine learning model. **(c)** Zoomed-in region of the embryo showing (top) few nuclei and RNAPII clusters, (middle) segmented clusters and (bottom) merged image. A representative Histone Locus Body (HLB) is marked by a pink arrow. The HLBs are excluded from the segmentation. Scale bar is 2 μm. Cluster segmentation was performed in 3D using a custom analysis pipeline (see Methods for more detail).
**Fig. S3.3: Gaussian-mixture model (GMM) fitting of cluster survival data to extract lifetimes for different populations**. **(a)** Survival curves for cluster lifetimes from nc11–14. 120 clusters were measured for each nuclear cycle from at least 3 embryos with at least 5 nuclei per embryo. Schematic on the right shows approximate interphase durations during the different nuclear cycles. **(b)** Fitting the probability density functions (PDFs) for the cluster lifetimes as a fraction of interphase length for the vehicle embryos in nc11–14. Dashed gray lines on each pdf represent the different gaussian fits. **(c)** Fitting the PDFs for the cluster lifetimes as a fraction of interphase length for the α-amanitin-injected embryos in nc13 (left) and nc14 (right). Dashed grey lines on each PDF represent the different Gaussian fits. Cluster lifetimes for triptolide-injected embryos could not be fit to a multi-Gaussian model, as only short-lived populations were observed.
**Fig. S4.1: Cluster calling validation and filtering. (a)** Representative spatial mapping of average single molecule trajectories (left) and the clusters that are identified on the same field of view using DBSCAN. Scale bar is 4 μm. **(b)** Representative elbow curve analysis to determine DBSCAN parameters. **(c)** Clusters were identified using the Voronoi tessellation method as an orthogonal approach to validate DBSCAN-based cluster detection. **(d)** Number of clusters identified using the two different cluster calling methods in the same embryo and same field of view. **(e)** RPB1 bound fraction inside the clusters determined by two different cluster calling methods in the same embryo and same field of view. **(f)** Probability density functions for the number of trajectories inside clusters in nc13 (left) and nc14 (right) for vehicle embryos (top row) and α-amanitin injected embryos (bottom row). Number of clusters are 1,517 and 202 for vehicle and α-amanitin injected embryos respectively in nc13 and 1,667 and 258 for the same conditions in nc14.
**Fig. S4.2: Anisotropy calculation and angle distribution. (a)** Histogram of jump distances from H2B trajectories in nc14 used to determine the distance threshold for anisotropy analysis. A total of 51,158 tracks were considered. **(b)** Schematic showing which angle is considered for the calculation for every pair of displacements. **(c)** Histograms showing angle distributions for different conditions in nc13 (left) and nc14 (right). Each row represents a different condition and the condition is labelled on the top of each plot in the left column. Number of angles in the vehicle case are 13,107, 3,070, and 5,754 for outside clusters, inside clusters and in control spots respectively in nc13 and 10,540, 2,057, and 6,104 for the same categories in nc14. Number of angles in the α-amanitin injected embryos are 2,664, 344 and 2,189 for outside clusters, inside clusters, and in control spots respectively in nc13 and 4,807, 448, and 2,894 for the same categories in nc14.
**Fig. S5.1: Measurement of RNAPII and MCP intensities around MS2 spots. (a)** Schematic showing the measurement of intensities around the MS2 spot (magenta star) in the nucleus. A 41×41 pixel area (black dotted line) around the MS2 spot was cropped for each time point imaged. The RPB1-GFP and the MCP-mCherry intensities in a 0.5 μm radius ROI centered at the MS2 spot (magenta dotted circle) at each time point were recorded. **(b)** Orthogonal view max projections for an example nucleus at a time point where the *hb*-MS2 reporter is actively transcribing, showing that the MS2 spot (magenta arrow) is not associated with HLBs (green arrows). Intensities for each orthogonal view were scaled independently. Scale bar is 1 μm.
**Fig. S5.2: Additional examples of RNAPII clusters remaining stably associated with an MS2 spot.** Orthogonal view images of nuclei in nc13 (top) and nc14 (bottom) with RPB1 (grey) and MCP-mCherry (pink) labelling active transcription at a *hb*-MS2 reporter during a ~15 min transcriptional burst. Cytoplasmic MCP signal is present as it is expressed without a nuclear localization signal.

Supplement 2 **Movie 1**: Example of single molecule tracking in a live embryo of (left) an RPB1-mEos3.2, (middle) His2B-mEos3.2, and (right) NLS-mEos4.0. In all cases, the movies shown here are over a 3 second period (i.e. 300 frames at 10 ms). All movies are sped up to 8 fps.

Supplement 3 **Movie 2:** Movie of an embryo showing hunchback MS2 spots in nc14 after vehicle injection in nc13. Each frame is the maximum intensity projection of a z-stack composed of 64 slices with 300 nm slice thickness and exposure time of 60 msec. The time interval between each stack is 10 sec. Movie is sped up to 8 fps.

Supplement 4 **Movie 3:** Embryo in nc14 showing that hunchback MS2 spots disappear after α-amanitin injection in nc13. Each frame is the maximum intensity projection of a z-stack composed of 64 slices with 300 nm slice thickness and exposure time of 60 msec. The time interval between each stack is 10 sec. Movie is sped up to 8 fps.

Supplement 5 **Movie 4:** Embryo in nc14 showing that hunchback MS2 spots disappear after triptolide injection in nc13. Each frame is the maximum intensity projection of a z-stack composed of 64 slices with 300 nm slice thickness and exposure time of 60 msec. The time interval between each stack is 10 sec. Movie is sped up to 8 fps.

Supplement 6 **Movie 5:** Volumetric imaging of RPB1-GFP in a vehicle injected embryo from nc10 till the end of nc13. This movie highlights the RPB1 clusters observed across the nuclear cycles. Each frame is the maximum intensity projection of a z-stack composed of 64 slices with 300 nm slice thickness and exposure time of 60 msec. The time interval between each stack is 10 sec. Movie is sped up to 8 fps.

Supplement 7 **Movie 6:** Volumetric imaging of RPB1-GFP in an α-amanitin injected embryo in nc14. The embryo was injected in nc12 and imaged from nc13 onwards. Each frame is the maximum intensity projection of a z-stack composed of 64 slices with 300 nm slice thickness and exposure time of 60 msec. The time interval between each stack is 10 sec. Movie is sped up to 8 fps.

Supplement 8 **Movie 7:** Volumetric imaging of RPB1-GFP in a triptolide injected embryo in nc14. The embryo was injected in nc12 and imaged from nc13 onwards. Each frame is the maximum intensity projection of a z-stack composed of 64 slices with 300 nm slice thickness and exposure time of 60 msec. The time interval between each stack is 10 sec. Movie is sped up to 8 fps.

Supplement 9 **Movie 8:** Example of tracked clusters in a vehicle injected embryo (single z-slice) for determination of average lifetime of clusters. Yellow circles indicate tracked clusters. Scale bar is 1 μm.

Supplement 10 **Movie 9:** Example of tracked clusters in an α-amanitin injected embryo (single z-slice) for determination of average lifetime of clusters. Yellow circles indicate tracked clusters. Scale bar is 1 μm.

Supplement 11 **Movie 10:** Example of tracked clusters in a triptolide injected embryo (single z-slice) for determination of average lifetime of clusters. Yellow circles indicate tracked clusters. Scale bar is 1 μm.

## Figures and Tables

**Table:** Key Resources

Reagent or Resource	Source	Identifier	Additional Information
**Fly Lines**			
RPB1-LL-eGFP	Eisen Lab (UC Berkeley)	-	The line was made using CRISPR-Cas9 system. The construct was made with 1x-FlagTag at the N-terminal of the eGFP. The eGFP is linked to the RPB1 with the long linker sequence: SGDSGVYKTRAQASNSAVDGTAGPGSTGSS.
His2B-eGFP	Mir et al. 2018	-	Fly line with an H2B-eGFP transgene inserted on chromosome 3. Transgene is expressed ubiquitously under the control of a synthetic tubulin promoter.
RPB1-LL-mEos3.2	Eisen Lab (UC Berkeley)	-	The line was made using CRISPR-Cas9 system. The construct was made with 1x-FlagTag at the N-terminal of the mEos. The mEos is linked to the RPB1 with the long linker sequence: SGDSGVYKTRAQASNSAVDGTAGPGSTGSS.
His2B-mEos3.2	Mir et al. 2018	-	Fly line with an H2BmEos3.2 transgene inserted on chromosome 3. Transgene is expressed ubiquitously under the control of a synthetic tubulin promoter.
NLS lines	This paper	-	Fly line with an NLS-mEos3.2 transgene inserted on chromosome 3. Transgene is expressed ubiquitously under the control of a synthetic tubulin promoter. NLS11: AAAKRSWSMAF was used^51^.
**Antibodies**			
Western Blot reagents	Abcam	ab5131	Anti-RNA polymerase II CTD repeat YSPTSPS (phospho S5) antibody
	Abcam	ab179513	Recombinant Anti-beta Tubulin antibody
**Microinjection Drugs**			
Triptolide	MedChemExpress	HY-32735	Transcriptional initiation inhibitor
α-amanitin	Sigma Aldrich	A2263-1mg	Transcriptional elongation inhibitor
DMSO	Cell Signaling Technologies	12611PP	
**Critical Commercial Reagents**			
Tetraspeck beads	Thermofisher	T7280	Beads for microscope calibration
Alexafluor dyes	Thermofisher	Alexa Fluor 488 Dye, Alexa Fluor 594 Dye	Dye for microscope calibration
Halocarbon Oil 27	Sigma-Aldrich	H8773-100ML	
n-Heptane	McMaster	3190K548	
**Software**			
Quot	https://github.com/alecheckert/quot	N/A	
saSPT	https://github.com/alecheckert/saspt	N/A	
Custom Hub Analysis, MS2-Hub Interaction Analysis	Zenodo	10.5281/zenodo.14834785	
Custom SMT Analysis	Zenodo	10.5281/zenodo.14834785	

## Data Availability

Compiled volumetric data are available on Zenodo: 10.5281/zenodo.14834785 (in the folder titled “Data”). SMT and MS2-Hub Interaction data are available on request.
