# Supplementary material for: A cluster of RNA Polymerase II molecules is stably associated with an active gene
Source: bioRxiv. 2025 Feb 11:2025.02.10.637507. Preprint. [Version 1] doi: 10.1101/2025.02.10.637507 (PMC11844394; doi:10.1101/2025.02.10.637507)
Supplement: Supplementary file 1 [file media-1.docx]

### Supplementary Figures

**Fig. S1.1. Constructs used for imaging. (a)** Both constructs were generated using the CRISPR-Cas9 system. Each construct includes a Flag-tag at the N-terminus of the fluorescent protein (eGFP or mEos3.2) and is linked to RPB1 via a long linker. **(b)** Western blots show that eGFP and mEos3.2-tagged RPB1 have similar expression levels to wild-type RPB1, as confirmed by tubulin loading control. The double bands in RPB1 blot correspond to previously reported phosphorylated and unphosphorylated states of the RPB1 C-terminal domain[^58^](https://paperpile.com/c/vRgHue/xigg1). 

**Fig. S1.2: Single molecule tracking quality control.** **(a)** Representative traces showing the number of detections per frame per nucleus in nc13 (top) and nc14 (bottom). **(b)** Analysis pipeline showcasing how the diffusion coefficient spectrum is divided into different kinetic states based on the local minima values. Local minima are represented by blue dots. The different kinetic states are shown as different shaded regions. **(c)** Heatmaps of the probability distributions of diffusion coefficients obtained for each movie (one field of view/row; 8-15 nuclei per field of view) for nc13 (top) and nc14 (bottom). 

**Fig. S2.1: Validation of the microinjection process.** **(a)** Schematic showing an overview of the different stages of the microinjection process. **(b)** Quantification of the microinjection survival assay for vehicle injections (left), triptolide injections (center) and α-amanitin injections (right). **(c)** Representative images of embryos prior to injection showing hunchback MS2 signal (example spots indicated by pink arrows). Injection with either triptolide or α-amanitin causes the MS2 spots to disappear but after injecting with the vehicle the MS2 spots remain. Late in nc14 (~45 minutes into nc14) the embryo injected with the vehicle continues to develop normally while both of the drug injected embryos show major developmental defects validating the injection process. **(d)** Raw nuclear mean intensity of RNAPII. Data points are individual embryos. For vehicle-injected embryos, n=5 in nuclear cycle 13 and n=3 in nuclear cycle 14. For triptolide- and α-amanitin-injected embryos, n=3 in each nuclear cycle. Error bars represent standard deviation. **(e)** Heatmaps of the probability distributions of diffusion coefficients obtained for the vehicle injection, triptolide injection and α-amanitin injection in nc13 (left column) and nc14 (right column). Each row represents one movie.

**Fig. S2.2: Comparison of vehicle-injected embryos and uninjected embryos shows no difference in the SMT kinetics.** **(a)** Diffusion coefficient distributions for the uninjected embryo compared to the vehicle embryo in nc13 (top) and nc14 (bottom). A total of 210,638 trajectories across 9 independent embryos and 205,532 trajectories across 10 independent embryos were obtained for nc13 and nc14 respectively for the vehicle case. A total of 203,697 trajectories across 10 independent embryos and 254,957 trajectories across 10 independent embryos were obtained for nc13 and nc14 respectively for the uninjected case. **(b)** Bound, intermediate, and fast fractions for both the uninjected and vehicle cases in nc13 (top) and nc14 (bottom) are shown. Individual data points represent individual movies and error bars represent standard deviation. 

**Fig. S3.1: RNAPII clusters are visible from nc10 to nc14.** Time-lapse snapshots showing five consecutive imaging time points (rows) during interphase for each nuclear cycle from nc10 to nc14 (columns). Image contrasts are all scaled equally. Images are maximum intensity projections of deconvolved images over 16.8 μm. Scale bar is 1 μm.

**Fig. S3.2: Characterization of RNAPII clusters.** **(a)** Volumes of a layer of nuclei on the *Drosophila* embryo surface are acquired during imaging and deconvolved. Scale bar is 12 μm. **(b)** Nuclei are segmented in 3D using a trained machine learning model. **(c)** Zoomed-in region of the embryo showing (top) few nuclei and RNAPII clusters, (middle) segmented clusters and (bottom) merged image. A representative Histone Locus Body (HLB) is marked by a pink arrow. The HLBs are excluded from the segmentation. Scale bar is 2 μm. Cluster segmentation was performed in 3D using a custom analysis pipeline (see Methods for more detail). 

**Fig. S3.3: Gaussian-mixture model (GMM) fitting of cluster survival data to extract lifetimes for different populations**. **(a)** Survival curves for cluster lifetimes from nc11-14. 120 clusters were measured for each nuclear cycle from at least 3 embryos with at least 5 nuclei per embryo. Schematic on the right shows approximate interphase durations during the different nuclear cycles. **(b)** Fitting the probability density functions (PDFs) for the cluster lifetimes as a fraction of interphase length for the vehicle embryos in nc11-14. Dashed gray lines on each pdf represent the different gaussian fits. **(c)** Fitting the PDFs for the cluster lifetimes as a fraction of interphase length for the α-amanitin-injected embryos in nc13 (left) and nc14 (right). Dashed grey lines on each PDF represent the different Gaussian fits. Cluster lifetimes for triptolide-injected embryos could not be fit to a multi-Gaussian model, as only short-lived populations were observed.

**Fig. S4.1: Cluster calling validation and filtering.** **(a)** Representative spatial mapping of average single molecule trajectories (left) and the clusters that are identified on the same field of view using DBSCAN. Scale bar is 4 μm. **(b)** Representative elbow curve analysis to determine DBSCAN parameters. **(c)** Clusters were identified using the Voronoi tessellation method as an orthogonal approach to validate DBSCAN-based cluster detection. **(d)** Number of clusters identified using the two different cluster calling methods in the same embryo and same field of view. **(e)** RPB1 bound fraction inside the clusters determined by two different cluster calling methods in the same embryo and same field of view. **(f)** Probability density functions for the number of trajectories inside clusters in nc13 (left) and nc14 (right) for vehicle embryos (top row) and α-amanitin injected embryos (bottom row). Number of clusters are 1,517 and 202 for vehicle and α-amanitin injected embryos respectively in nc13 and 1,667 and 258 for the same conditions in nc14.

**Fig. S4.2: Anisotropy calculation and angle distribution.** **(a)** Histogram of jump distances from H2B trajectories in nc14 used to determine the distance threshold for anisotropy analysis. A total of 51,158 tracks were considered. **(b)** Schematic showing which angle is considered for the calculation for every pair of displacements. **(c)** Histograms showing angle distributions for different conditions in nc13 (left) and nc14 (right). Each row represents a different condition and the condition is labelled on the top of each plot in the left column. Number of angles in the vehicle case are 13,107, 3,070, and 5,754 for outside clusters, inside clusters and in control spots respectively in nc13 and 10,540, 2,057, and 6,104 for the same categories in nc14. Number of angles in the α-amanitin injected embryos are 2,664, 344 and 2,189 for outside clusters, inside clusters, and in control spots respectively in nc13 and 4,807, 448, and 2,894 for the same categories in nc14.

**Fig. S5.1: Measurement of RNAPII and MCP intensities around MS2 spots.** **(a)** Schematic showing the measurement of intensities around the MS2 spot (magenta star) in the nucleus. A 41x41 pixel area (black dotted line) around the MS2 spot was cropped for each time point imaged. The RPB1-GFP and the MCP-mCherry intensities in a 0.5 μm radius ROI centered at the MS2 spot (magenta dotted circle) at each time point were recorded. **(b)** Orthogonal view max projections for an example nucleus at a time point where the *hb*-MS2 reporter is actively transcribing, showing that the MS2 spot (magenta arrow) is not associated with HLBs (green arrows). Intensities for each orthogonal view were scaled independently. Scale bar is 1 μm.

**Fig. S5.2: Additional examples of RNAPII clusters remaining stably associated with an MS2 spot.** Orthogonal view images of nuclei in nc13 (top) and nc14 (bottom) with RPB1 (grey) and MCP-mCherry (pink) labelling active transcription at a *hb*-MS2 reporter during a ~15 min transcriptional burst. Cytoplasmic MCP signal is present as it is expressed without a nuclear localization signal.
